# 12 Tips to Improve ENT Training During the Covid-19 Pandemic

**DOI:** 10.15694/mep.2021.000059.1

**Published:** 2021-03-01

**Authors:** Dilhara Karunaratne

**Affiliations:** 1 Eastbourne District General Hospital

**Keywords:** Telephone clinic, feedback, simulation, webinar, flexible nasoendoscopy

## Abstract

This article was migrated. The article was marked as recommended.

**Background:** The Covid-19 pandemic has resulted in significant change to ENT practice, as it a high-risk speciality in terms of SARS-Cov-2 virus transmission. The reduction in theatre time, loss of face to face outpatient appointments and redeployment has contributed to significant loss of training opportunities for current ENT trainees.

**Aim:** This paper provides twelve easy and simple tips for current ENT trainees to follow.

**Methods:** Relevant literature was reviewed and the first named author’s personal experiences were drawn upon.

**Results:** The twelve tips are (1) Try run a telephone clinic, (2) Evaluate face to face patients independently and make a management plan, (3) Gain formative feedback, (4) Make the ward round a learning opportunity, (5) Organise formal consultant led teaching, (6) Learn from the interprofessional team, (7) Attend online courses and webinars, (8) Take pictures when performing flexible nasoendoscopy , (9) Organise simulation sessions, (10) Gain surgical knowledge and exposure outside of the theatre setting, (11) Undertake an audit or quality improvement project and (12) Look after yourself and make mental health a priority

**Conclusion:** These twelve tips should help the ENT trainee to maximise their learning opportunities and improve their training.

## Introduction

The Covid-19 pandemic had led to significant changes in medical practice across the world. Within the United Kingdom, several steps were taken to optimise the medical workforce to combat the pandemic. Some of the first measures to be taken was reduction of direct patient contact by running outpatient clinics with telephone consultation in the first instance. Elective surgery was cancelled, with only emergency and cancer surgery ongoing. Surgical junior doctors were redeployed from their parent speciality into intensive care or medical specialities, to help with the volume of unwell Covid-19 patients.

Surgical training was adversely affected by these changes, with trainees missing out on vital practical experience and clinical exposure. Within surgery, ENT was an especially risky speciality, due to its direct contact with areas of the body such as the oral cavity and nasal cavity that the SARS-Cov-2 virus infects ( Zou *et al.,* 2020). Due to this, clinical contact with patients was reduced as much as was possible. Therefore, ENT trainees particularly are now in a vulnerable position with regards to training. This article focuses on my experience as a CT2 ENT trainee and the steps I have taken in order to improve my education and to maximise my learning experiences. The pandemic is yet ongoing and the changes in surgical practice are here to stay, therefore it is vital that we adapt to this new way of training and make the most of what is available to us currently. My tips are simple and easy to follow and should be of value to current surgical trainees in a similar predicament.

## Tip 1

### Try running a telephone clinic

ENT is a speciality with large outpatient clinic volume as many ENT conditions can initially be managed medically rather than surgically. Therefore, the introduction of telephone clinic has had a big impact on the functionality of the speciality. Telephone clinics may not initially seem useful for training, especially if there is no speaker function on the phone to allow you to listen in on the consultation. Having initially been disheartened by the lack of face to contact, I was determined to make it of educational value however possible.

I found that telephone clinic is a useful way to learn focused history taking and to weigh up the conversation and decide whether a patient needs a face to face consultation, whether a further telephone conversation is appropriate or whether discharge is suitable. I now sit it in consultant clinic and do the telephone consultations as much as I can, under their guidance and observation. In this way, I have improved my history taking skills and am good at asking about red flag symptoms in a systematic way. It has also improved my communication skills, as patient information and reassurance needs to delivered using words, without the aid of body language. There is evidence to suggest that patients are satisfied with telephone appointments ( Wolthers and Wolthers, 2020) and they may remain in the post Covid era, so it is vital they we adapt to them and maximise their usefulness.

My recommendation would be to run as many telephone appointments as possible, and if you decide to see the patient face to face, to try and arrange the follow up with yourself in order to improve continuity of care and the learning potential from the patient’s pathology.

## Tip 2

### Evaluate face to face patients independently and make a management plan

The reduction in volume of face to face patients has made gaining experience in the outpatient setting difficult. Therefore, it is important to see face to face patients and take the opportunity to examine them whenever possible. At the beginning of each day, I take a look at clinic lists and see whether any patients are face to face and note down the time that are due to be seen. I then make the time to attend the clinic to see the patient in between theatre cases or ward work. I ask the consultant whether I could see the patient myself, and then do an independent history taking, examination and management plan and then present this to the consultant. As the consultants are less time constrained in clinics owing to the reduction in numbers of face to face patients, I have found that they have the time to discuss the case in detail with me, and therefore provide me with a rich learning experience.

## Tip 3

### Gain formative feedback

Following on from tips 1 and 2, gaining formative feedback from a consultant can be a great opportunity for constructive criticism, self-reflection and continuing professional development. There is evidence to suggest that feedback improves doctors’ skillsets and establishes life-long learning ( Kelly and Richards, 2019), and given the limited patient contact and learning opportunities due to Covid-19, making time for more formative feedback opportunities may be key in training. I try to make formative feedback opportunities as much as a I can and often ask my seniors on what I did well, what I did not do well and what I can improve. These feedback sessions can be formally put in writing and logged on your portfolio, which can then provide evidence of your engagement in training and commitment to improvement.

## Tip 4

### Make the ward round a learning opportunity

Ward rounds can be busy environments and the consensus appears to be that they are more tailored towards service provision than teaching ( Laskaratos, Parry and El-Mileik, 2016), making them a wasted educational opportunity. Within my trust, I have observed that surgical inpatient admission numbers have declined during the Covid-19 pandemic and this allows the clinicians time to discuss cases in more detail. In my institution we have made time during ward rounds to learn from the inpatients, to discuss their history, examination, radiology and management plan in detail. This is valuable for surgical trainees to learn decision making which will be useful as they progress to higher training.

## Tip 5

### Organise formal consultant led teaching

Although the literature suggests that consultants enjoy teaching and deem it important in medical education, time is a major constraint in organising formal consultant led teaching ( Darragh, Baker and Kirk, 2015). Given the reduction of clinic and inpatient volumes within ENT in my own institution, I have made the most of increased consultant availability by arranging formal weekly teaching. Requiring a degree of proactivity on my part, I pick a suitable topic and make a presentation on it and deliver the presentation to my team within a socially distanced setting. Over an hour or so, we discuss the topic with a consultant. This makes for a great educational opportunity for the entire team and sharing of knowledge and ideas.

## Tip 6

### Learn from the interprofessional team

Following on from tip 5 which advised to gain knowledge from consultants, tip 6 focuses on gaining a different experience from the interprofessional team. ENT teams often work closely with specialist nurses, audiologists and speech and language therapists and prior to Covid-19 I have personally not had a lot of contact with them, and therefore did not have a broad understanding of their field. Interprofessional learning is valuable in healthcare, and is shown to improve patient outcomes ( Winter, Ingledew and Golden, 2019), so taking this opportunity to spend time with allied healthcare professionals is important. During my current training, I have spent time with the audiology team to understand how exactly they conduct hearing tests and also improved my understanding of the functionality of different types of hearing aids. With the speech and language therapy team, I have broadened my knowledge and examination skills of swallowing disorders and observed how they undertake a functional endoscopic examination of swallowing. I have also spent time with the head and neck specialist nurse and gained valuable communication skills in the observation of the nurse’s day to day practice and support of oncology patients during the pandemic. In this way, interprofessional education can be helpful in producing a well-rounded trainee, with a broad set of skills.

## Tip 7

### Attend online courses and webinars

The cancellation of educational courses has been to the dismay of many and the inability to deliver certain types of courses within a socially distanced setting have led to them being delivered virtually. There are many webinars being advertised on medical topics which are well worth attending and I have found that the increase in courses being delivered virtually has allowed me to virtually attend courses running in other countries, that I probably otherwise would not have attended owing to financial and study leave constraints. In this way, Covid-19 has been useful in improving the way we use technology and sharing information and is allowing access to education internationally, which trainees can benefit from.

## Tip 8

### Take pictures when performing flexible nasoendoscopy

Flexible nasoendoscopy is commonly undertaken ENT procedure, both in the emergency and elective setting. Due to its aerosol generating risk, it is now undertaken only when necessary and with the minimal numbers of staff present. This has led to a reduction in learning opportunities for the ENT trainee, particularly in the identification of abnormalities. In my institution, we have a video stack system which allows for videos or photos to be taken of the flexible nasoendoscopy procedure, which then allows for the consultant to discuss the findings with the trainees and thereby provide a learning experience even if the trainee was unable to observe the procedure directly.

## Tip 9

### Organise simulation sessions

Simulation is now a significant component of medical education, allowing the trainee to pick up technical and non-technical skills in a non-threatening environment ( Khaliq and Atif, 2019). I have recently attended a tracheostomy simulation session where I was exposed to clinical scenarios involving tracheostomy emergencies such as airway obstruction or tracheostomy bleed. Given the reduction in clinical ENT exposure during the Covid-19 pandemic, this simulation session was extremely valuable in improving my confidence in managing such emergencies. Recent literature also shows innovative techniques in simulating aural microscopy skills and myringotomy and grommet insertion using simple tools ( Shenton and Aucott, 2020), which are useful in gaining and maintaining existing skills. In this way, simulation can be valuable adjunct for training during this period.

## Tip 10

### Gain surgical knowledge and exposure outside of the theatre setting

I have found the Emergency Department a valuable place to practice my suturing skills, as my time in the operating theatre has been limited. Being proactive and approaching the Emergency Department staff and letting them know of your interests can help you become involved in the suturing of minor lacerations under local anaesthetic and improve your surgical skills. I have also used online videos of surgical procedures in order to learn surgical steps and techniques and sometimes watch it with a consultant in order to discuss the steps in further detail, which aids my understanding and memory.

## Tip 11

### Undertake an audit or quality improvement project

If you find that you have some extra time, it is a good idea to undertake a project. Projects of good quality can then be submitted for presentation or publication and thereby enhance your CV. You may also help your hospital in standardising care by auditing the changes to clinical practice resulting from Covid-19 and looking at patient outcomes.

## Tip 12

### Look after yourself and make mental health a priority

This is an extremely difficult time for medical professionals, with evidence to suggest higher levels of stress and burnout during the management of the Covid-19 pandemic ( Kannampallil *et al.,* 2020). Taking time for yourself and engaging in wellness strategies is important during this time to maintain your mental health and it will have the added benefit of increasing your motivation and productivity.

## Conclusions

The Covid-19 pandemic has brought about unprecedented changes to clinical practice, with ENT significantly affected as it is a high-risk speciality. With the pandemic still ongoing and no plans for clinical practice to return to normal, it is important that ENT trainees learn to adapt to the current situation. The above twelve tips are simple and should be easy to follow and will help the ENT trainee maximise their learning opportunities. I believe that regular practice of these tips will allow the core ENT trainee to safely enter higher training.

## Take Home Messages

• The Covid-19 pandemic has brought about significant changes to ENT practice in the UK.
• Enthusiasm, a degree of proactivity and maximising existing training opportunities should help the current ENT trainee in achieving an improved training experience.
• The above tips are simple and easy to follow and should help mitigate the current changes to training structure and reduction of opportunities.

## Notes On Contributors

Dilhara Karunaratne is a core surgical trainee in ENT and has a strong interest in medical education. She graduated with honours in medicine from the University of Birmingham in 2016, also with an intercalated honours degree from King’s College London in physiology in 2014. She is currently undertaking a diploma in clinical education with Brighton and Sussex Medical School.

ORCiD: https://orcid.org/0000-0003-0705-7176
