# Supplementary material for: 12 Tips to Improve ENT Training During the Covid-19 Pandemic
Source: MedEdPublish (2016). 2021 Mar 1;10:59. [Version 1] doi: 10.15694/mep.2021.000059.1 (PMC10939616; doi:10.15694/mep.2021.000059.1)
Supplement: Peer review history

# Reviewer report: Reviewer response for version 1

**DOI:** 10.21956/mep.18936.r27086

**Published:** 2021-03-16

**Authors:** Megan Anakin

**Affiliations:** 1 University of Otago

This review has been migrated. The reviewer awarded 3 stars out of 5 I came across your article because I am supervising an ENT trainee who is completing a Master’s degree on the topic of ENT education, therefore, we read and discussed your article. My review includes suggestions that arose due to our conversation about your article.It is refreshing and very helpful to read an article from the learner’s point of view. My comments are directed at enhancing the scholarship of this article and so it will provide deeper insights to learners and educators who are keen to support ad further develop ENT education. Please consider revising the headings used in your abstract so it better reflects the structure of your article. Currently the headings in the abstract are typically used to signpost a research article, whereas, three headings such as introduction, tips, and conclusion might better introduce the reader to the main focus of your practical tips article.Please support the claims made in the second, third, fourth, and fifth sentences of the introductory paragraph with references. Please explain why you think that “the changes in surgical practice are here today”. Please consider revising the last sentence of this paragraph because by stating “My tips are simple and easy to follow” does not necessarily make them so for an international reader who is learning or supervising in a different context. Please consider softening this statement to acknowledge that others might find some of your tips challenging to implement in their circumstances by stating something like: “These tips are intended to be simple and easy to follow… As done with seven of the 12 tips, include at least one scholarly reference to support the claims made about the features of each tip. The use of examples to illustrate tips 1 and 6 are particularly vivid and helpful. Please consider reviewing the other tips to that they contain similarly illustrative examples for the reader. For example, for tip 2, what might a trainee do if consultants are not “less time constrained in clinics”? For tip 3, how might a trainee go about arranging a feedback session and what features might they be hoping to include in a good quality formative feedback session? In tip 4 please explain the term “trust” to an international reader. Please consider what tips 4 and 5 might like if surgical inpatient admission numbers have not declined – this exercise might help you identify the key features that should be part of ward round and formal consultant-led learning opportunities that could be enhanced with more time available but fewer patients. In tip 7, please consider replacing the term ‘virtual’ with ‘online’ in Tip because these terms are not synonyms.For tips 8-12, please consider further describing and explaining how you and your peers might use each tip in your local context then offer suggestions about how other might apply the tip to their situation.I would be very happy to read and review a revised version of this article.

Reviewer Expertise:

NA

No decision status is available

# Reviewer report: Reviewer response for version 1

**DOI:** 10.21956/mep.18936.r27085

**Published:** 2021-03-08

**Authors:** Anita Samuel

This review has been migrated. The reviewer awarded 4 stars out of 5 This article presents 12 tips for ENT trainees to proactively improve their ENT training during the pandemic. The tips presented are drawn from the personal experiences of the author, an ENT trainee. The tips are practical and accessible and also supported by the literature. Some points to consider:• The author’s proactive approach is commendable. However, it does depend on cooperative consultants. How do/did you get buy-in from the consultants?• Tip #1: How did you get to the position of being able to do telephone consultations? Are your consultants able to listen to your conversation? This is a great tip and other trainees might find it useful to know how to get to the position to conduct telephone consultations. • Tip #2: You mention making the time to attend the clinic in between theatre cases and ward work. How easy is it to balance this? Can you share how many you have done so others have a sense of what to expect?• Tip #5: You call this consultant led teaching and yet it looks like you select the topic and make a presentation. The consultant is only involved in the discussion stage. Can this really be called ‘consultant led’ teaching?• Tip #11 discusses projects. Can you provide examples of what you identify as ENT projects?• The tips you have provided can also be addressed to administrators as strategies they can implement for their trainees. You might want to mention the broader scope in the conclusion. • Are these tips limited to ENT or can they be applied to other specialties? • Will these tips have relevance after the pandemic when things return to normal? Can these be new ways to enhance learning?This is an interesting article which can be strengthened with a few revisions.

Reviewer Expertise:

NA

No decision status is available
